# Three-dimensional evaluation of pulp chamber volume and dimensions across skeletal and dental malocclusions using CBCT: a retrospective cross-sectional study

**DOI:** 10.1007/s00784-025-06568-y

**Published:** 2025-10-16

**Authors:** Raidan Ba-Hattab, Abeer Tamr, Muna Shaweesh, Shikha Jassim Alabduljabbar, Elham S. Abu Alhaija

**Affiliations:** 1 Pre-Clinical Oral Health Sciences Department, College of Dental Medicine, QU Health, Qatar University, Doha, Qatar; 2 Private Practice, Doha, Qatar; 3 Al Thumamah Health Center, Primary Health Care Corporation, Doha, Qatar; 4 Clinical Oral Health Sciences Department, College of Dental Medicine, QU Health, Qatar University, Doha, Qatar

**Keywords:** Pulp dimensions, Pulp volume, Dental malocclusion, Skeletal malocclusion, Cone-beam computed tomography

## Abstract

**Aim:**

To evaluate and compare pulp chamber volume(PCV) and dimensions of maxillary and mandibular first molars, and maxillary central incisors, among subjects with varying vertical and anteroposterior dental and skeletal malocclusions using cone beam computed tomography(CBCT).

**Materials and methods:**

A retrospective, cross-sectional analysis was conducted using CBCT scans from 184 Jordanian adults(mean age 24.22 ± 7.50 years). Subjects were categorized according to vertical facial pattern(short, average, and long face) and molar interdigitations (cusp-to-fossa, CFM; cusp-to-cusp, CCM). PCV and dimensions were measured using ITK-SNAP software. Data were analyzed with non-parametric tests and multiple linear regression.

**Results:**

For molars, PCV was significantly smaller in short-faced individuals than in average and long-face groups(*P* ≤ 0.001 across jaws), while average vs. long face was nonsignificant. In incisors, PCV differed among all three vertical groups (*P* = 0.001). Enamel thickness and crown height were also reduced in this group. CCM was associated with smaller PCV and reduced enamel and crown dimensions. Incisor PCV did not significantly differ by tooth contact status. Age, gender, molar interdigitation, and vertical pattern(for incisors) were significant predictors of PCVs (*P* ≤ 0.05).

**Conclusion:**

PCV and dimensions are influenced by vertical skeletal pattern, with short-faced individuals showing the smallest volumes. Molar interdigitation affects molar PCV, with CCM linked to reduced volumes. PCV showed a linear inverse association with age and are generally larger in males. Posterior crossbite correlates with increased molar PCV.

**Clinical relevance:**

Skeletal and occlusal variation should be considered during endodontic access planning and when interpreting radiographs to avoid misjudging pulp size in patients with atypical skeletal and dental relationships.

## Introduction

Occlusal forces and contact areas are key indicators in evaluating occlusal functionality [1]. In normal molar occlusion, the cusps of the maxillary and mandibular teeth are properly aligned with the fossae of the opposing arch, enabling occlusal forces to be transmitted vertically along the long axis of the teeth [2] Studies have shown that individuals with Class II and Class III malocclusions exhibit lower maximum bite force (MBF) values compared to those with normal Class I occlusion [3].

The presence of malocclusion may lead to the emergence of primary occlusal trauma, which is caused by an excessive force that acts on the healthy periodontium. It is found that the half-cusp class II relationship has more contact area than the full-cusp class II relationship as the palatal cusp is seated on the central fossa of the mandibular molar, while the palatal cusp in the full cusp class II relationship is sitting on the marginal ridge of the mandibular molar [4].

The pulp tissue enters the root canal as a large bundle of blood vessels, nerves, and connective tissue, maintaining continuity between the periapical tissues and the root canal space [5]. In response to occlusal trauma, dental pulp exhibits an increase in substance P, which initiates a neurogenic inflammatory process. This process involves the formation of new blood vessels, essential for stimulating mineralized tissue formation as a protective mechanism [6].

The influence of dental malocclusion on pulp morphology remains relatively underexplored. Individuals with an anterior open bite often exhibit a long facial profile, which has been associated with reduced BF [7]. Recent clinical evidence indicates that occlusal hypofunction, which is a characteristic of anterior open bite cases, may result in an increase in dental pulp volume (PV) [8]. This suggests a potential link between diminished occlusal loading and structural changes within the dental pulp, underscoring the need for further research into the biological implications of malocclusion beyond its effect on occlusal force.

To the best of our knowledge, no previous studies have investigated pulp chamber volume (PCV) and dimensions in relation to different anteroposterior and vertical dental malocclusions. To address this gap in the literature, the present study aimed to assess and compare the PCV and dimensions of maxillary and mandibular first molars and maxillary central incisors among individuals with varying vertical and anteroposterior skeletal and dental discrepancies.

## Materials and methods

### Study design

Retrospective, cross-sectional study.

This study was conducted in accordance with the Declaration of Helsinki, and received approval from the Research Ethical Committee (IRB) of Jordan University of Science and Technology (JUST) under reference number 2022/147/3. CBCT images, originally acquired for routine diagnostic purposes at JUST between January 2017 and January 2023, were retrospectively analyzed. As the data were fully anonymized and no additional interventions were performed, the requirement for informed consent was waived by the ethics committee.

All patients were Jordanians with a mean age of 23.95 ± 7.07 years (ranging from 18 to 40 years). Inclusion criteria were: age ≥ 18 years, healthy maxillary central incisors and first molars (no endodontic or restorative treatment and no signs of tooth wear, no history of trauma, no dental anomalies), no previous orthodontic treatment, and no history of any systemic diseases. Gender, age, anteroposterior molar relationship, posterior crossbite, and incisor relationship were recorded.

A total of 184 subjects (112 females and 72 males) were included in the study and subdivided based on vertical and anteroposterior molar relationships as follows:

### Vertical relationship

Subjects were divided into three groups based on the maxillary/mandibular (Max/Man) plane angle, defined as the angle between the palatal plane (anterior nasal spine to posterior nasal spine) and the mandibular plane (gonion to gnathion), measured on CBCT-reconstructed lateral cephalograms [9, 10], as follows:

#### Group 1: Short-faced subjects (SF)

The Max/Mand plane angle averaged 19.6 (± 3.1) degrees. It included 41 subjects (148 molars) distributed as follows: 41 maxillary right, 33 maxillary left, 36 mandibular, and 38 mandibular right first molars.

#### Group 2: average face subjects (AF)

The Max/Mand plane angle averaged 28.3 (± 2.6) degrees. It included 90 subjects (335 molars) distributed as follows: 90 maxillary right, 89 maxillary left, 85 mandibular left, and 71 mandibular right first molars.

#### Group 3: long face subjects (LF)

The Max/Mand plane angle averaged 35.8 (± 3.3) degrees. It included 53 subjects (194 molars) distributed as follows: 53 maxillary right, 50 maxillary left, 43 mandibular left, and 48 mandibular right first molars.

### Anteroposterior molar relationship

Posteriorly in the first molar region, subjects were classified into two groups based on CBCT occlusal reconstructions obtained with the mandible in maximum intercuspation (image-based assessment), as follows:

#### Group 1: Cusp-to-fossa relationship (CFM)

It included 116 subjects in which the functional cusp tip of the maxillary first molar seats in the central fossa of the opposing mandibular first molar (and vice versa), encompassing Class I, II, and III relationships (441 molars: 116 maxillary right, 111 maxillary left, 112 mandibular left, 102 mandibular right).

#### Group 2: Cusp-to-cusp (CCM)

It included 68 subjects in which opposing functional cusps contact, or a functional cusp opposes a marginal ridge, encompassing half-, three-quarter-, and quarter-unit Class II/III relationships (236 molars: 68 maxillary right, 61 maxillary left, 52 mandibular left, 55 mandibular right).

Anteriorly, pulp volume and dimensions of the maxillary incisors (366 maxillary incisors) were assessed based on vertical skeletal pattern and incisal contact. Incisors were categorized into: *Group 1*: Maxillary and mandibular incisors in contact during occlusion (223 central incisors), and *Group 2*: No contact between maxillary and mandibular incisors (143 central incisors).

### CBCT images

All CBCT images were taken using the same machine and operator. Imaging were taken using the KODAK 9500 cone beam 3D system (Carestream, Rochester, NY, USA), equipped with a flat panel detector. A medium field of view (FOV) measuring 15 cm in height and 9 cm in diameter was selected to capture the region of interest. Acquisition parameters were 90 kV, 10 mA, and an exposure time of 8.01 s. Each scan was obtained at an isotropic voxel size of 0.2 mm^3^. Patients were positioned upright with the Frankfort horizontal plane parallel to the ground as the scanner rotated 360° around the head.

### CBCT analysis

The CBCT images were independently evaluated by two experienced dentists (A.T. and M.S.), each with over 15 years of clinical practice. All assessments were conducted under standardized conditions for the teeth analyzed. To evaluate inter-examiner and intra-examiner reliability, 20% of the sample was re-assessed after a three-week interval and intraclass correlation coefficients has been analysed. Any discrepancies between examiners regarding molar relationship were resolved through consensus following individual evaluations.

Pulp dimensions and volumes were precisely measured using ITK-SNAP, a tool known for its accuracy in segmenting and analyzing 3D medical images [11] as follows:

### First molars’ pulp chambers dimensions and volumes

**Fig. 1:**
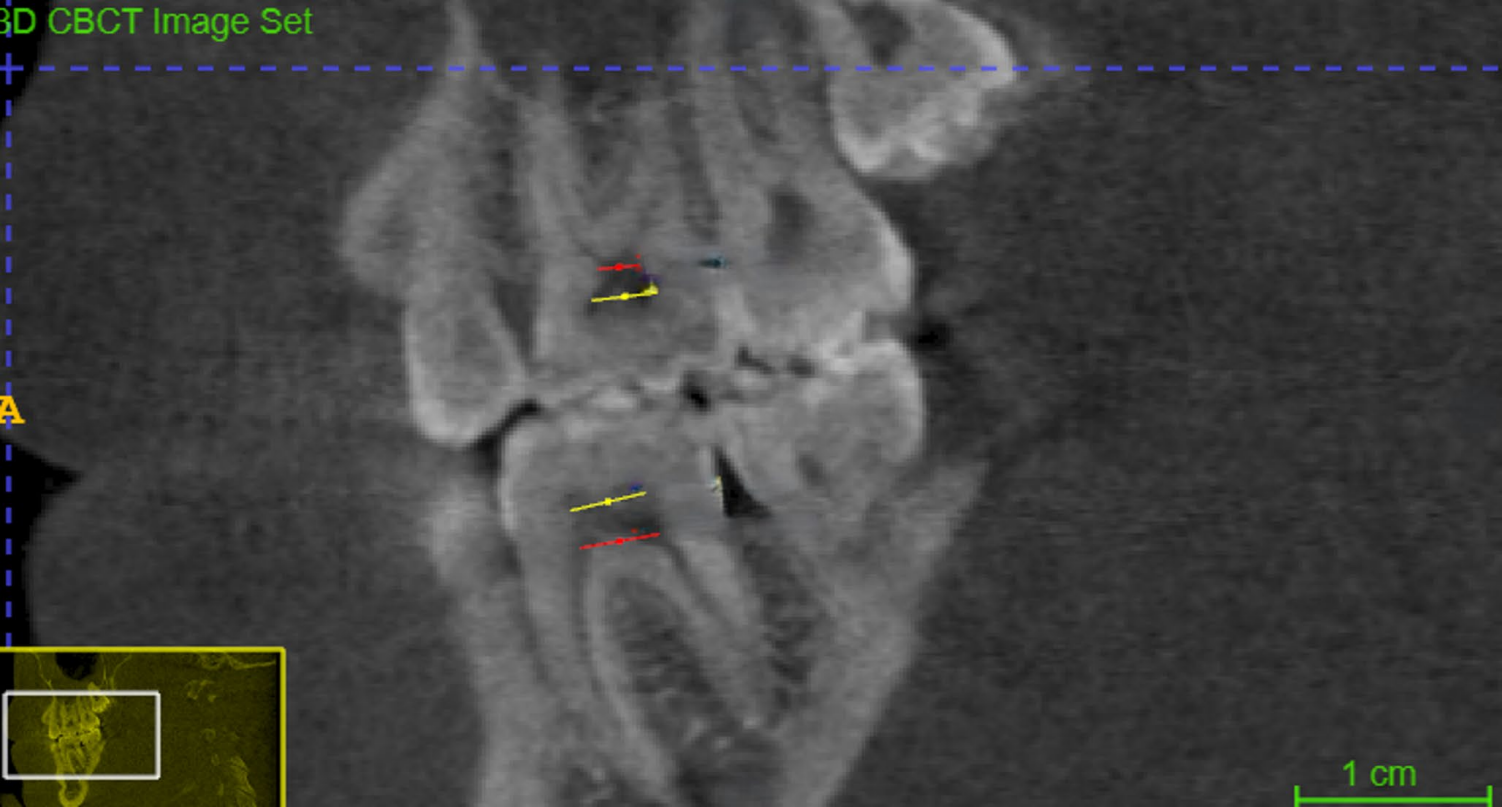
Pulp chamber width (PCW) in the sagittal section. The red line (PCW-F) represents the distance from the most mesial point to the most distal point of the floor of the pulp chamber. The yellow line (PCW-R) represents the distance from the most mesial point to the most distal point of the roof of the pulp chamber

*Pulp chamber width (PCW) *was measured in the sagittal view at two levels: PCW-F from the most mesial point to the most distal point at the floor of the pulp chamber, and PCW-R the same measurement at the roof of the pulp chamber (Fig. 1).

**Fig. 2:**
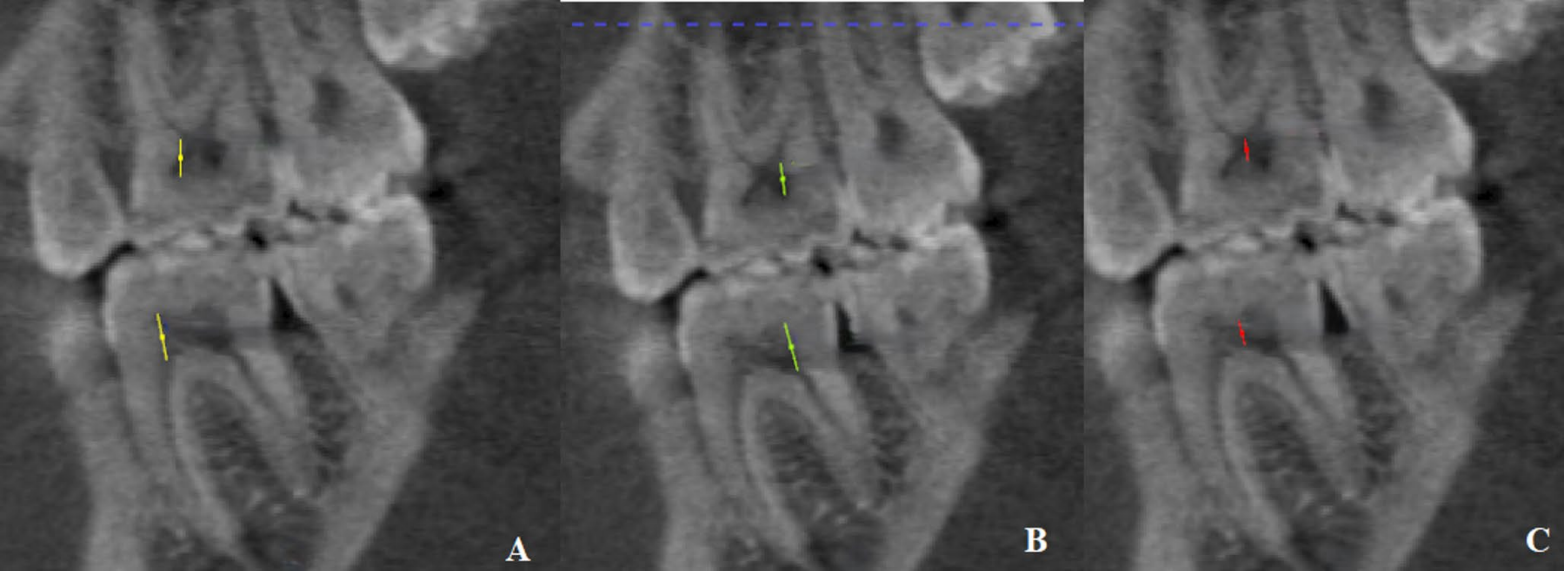
Pulp chamber height (PCH) in the sagittal view. (**A**) PCH-Mesial: the maximum distance from the floor of the pulp chamber to the tip of the mesial pulp chamber horn. (**B**) PCH-Distal: the maximum distance from the floor of the pulp chamber to the tip of the distal pulp chamber horn. (**C**) PCH-Middle: the maximum distance from the floor of the pulp chamber to the roof of the middle portion of the pulp chamber

*Pulp chamber height (PCH)* was measured in the sagittal view at three points (Fig. 2): PCH-Mesial, from the floor of the pulp chamber to the tip of the mesial pulp horn; PCH-Distal, from the floor to the tip of the distal pulp horn; and PCH-Middle, from the floor to the roof of the middle portion of the pulp chamber.

**Fig. 3:**
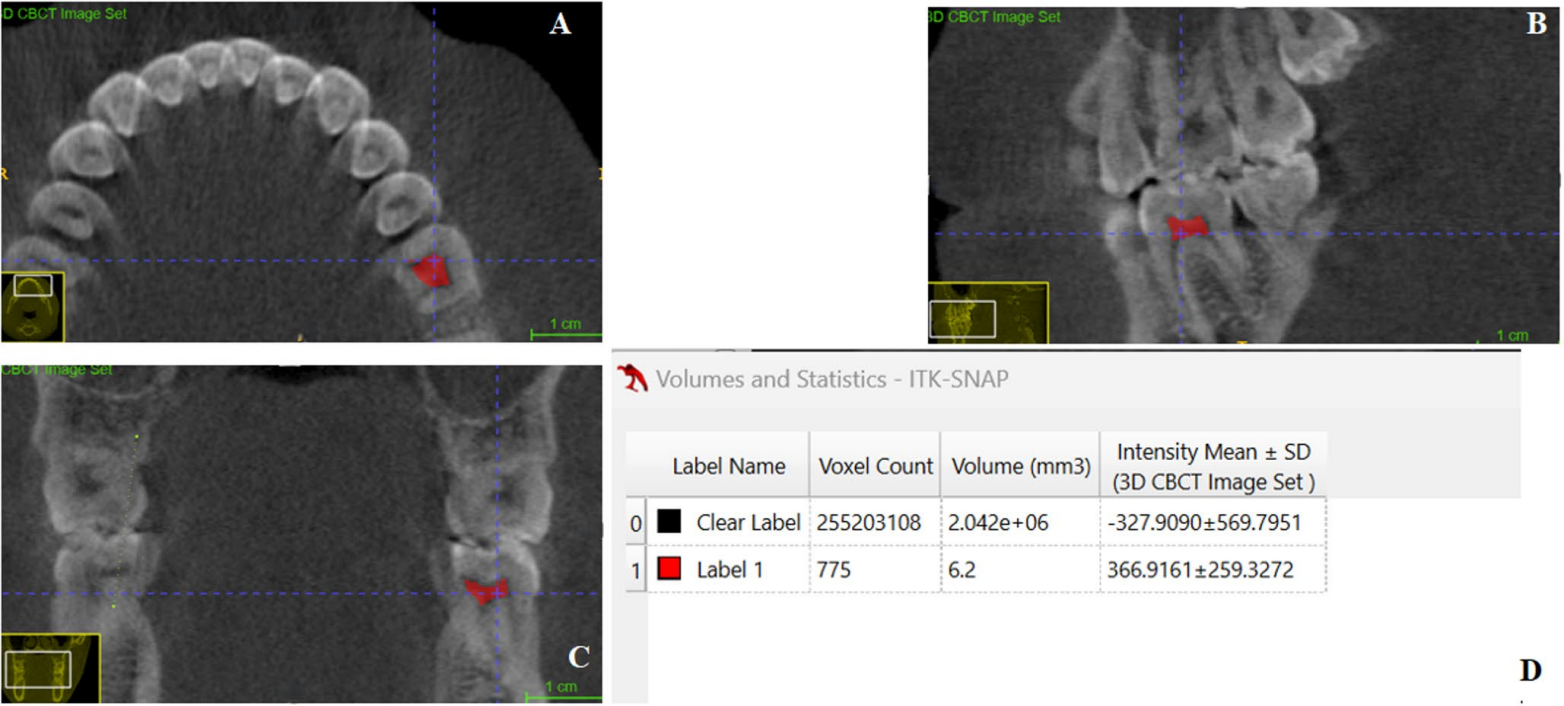
Pulp Chamber Volume (PCV). (**A**) PCV visualized in the axial plane, (**B**) PCV visualized in the sagittal plane, (**C**) PCV visualized in the coronal plane, (D) Volumetric analysis using the Volume and Statistics tool in ITK-SNAP software

*Pulp chamber volume (PCV)* The outline of the pulp chamber as visualized in the axial, sagittal, and the coronal planes (Fig. 3).

*Enamel thickness (En-Thick)* was measured in the sagittal view at two points: En-thick-Mesial and En-thick-Distal, as the distance from the dentino-enamel junction to the cusp tip over the mesial and distal pulp horns, respectively.

*Dentin thickness (Dent-Thick) *was measured in the sagittal view at two points: Dent-thick-Mesial and Dent-thick-Distal, as the distance from the dentino-enamel junction to the cusp tip over the mesial and distal pulp horns, respectively.

Mesiodistal crown width (MDCW): measured from mesial to distal at two levels in the sagittal view: at the roof (MDCW-R) and floor (MDCW-F) of the pulp chamber.

Crown height (CH): measured from the cementoenamel junction (CEJ) to the cusp tip, at mesial (CH-Mesial) and distal (CH-Distal) points in the sagittal view.

### Maxillary central incisors

**Fig. 4:**
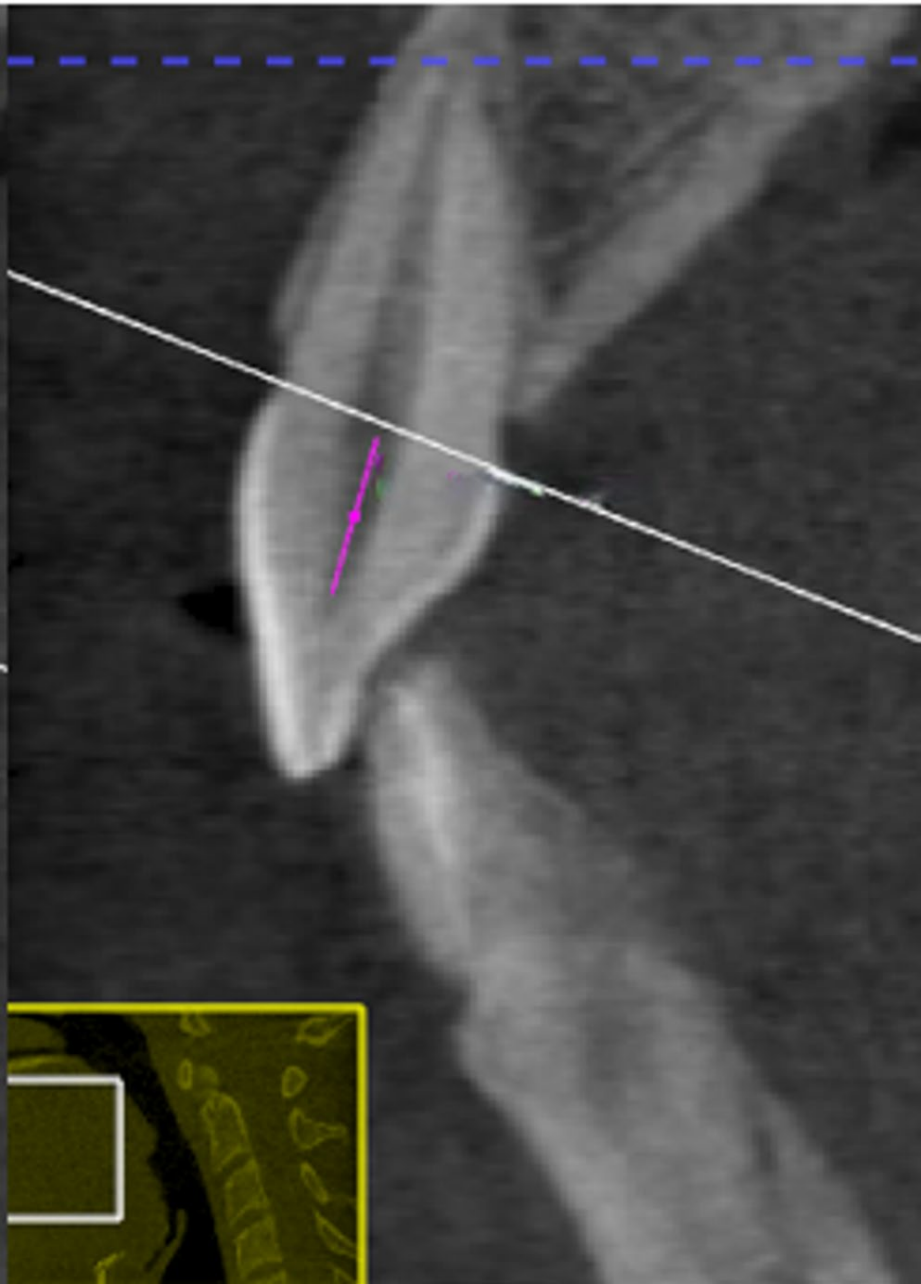
Maxillary Central incisor coronal pulp height (MICPH). The pink line represents the measurement of pulp chamber height from pulp at the level of cementoenamel junction to the most incisal point of the pulp chamber

*Maxillary incisor pulp chamber height (MICPH)* measured as the vertical distance from the top of the pulp chamber to its apical end within the crown portion, in the sagittal view (Fig. 4).

**Fig. 5:**
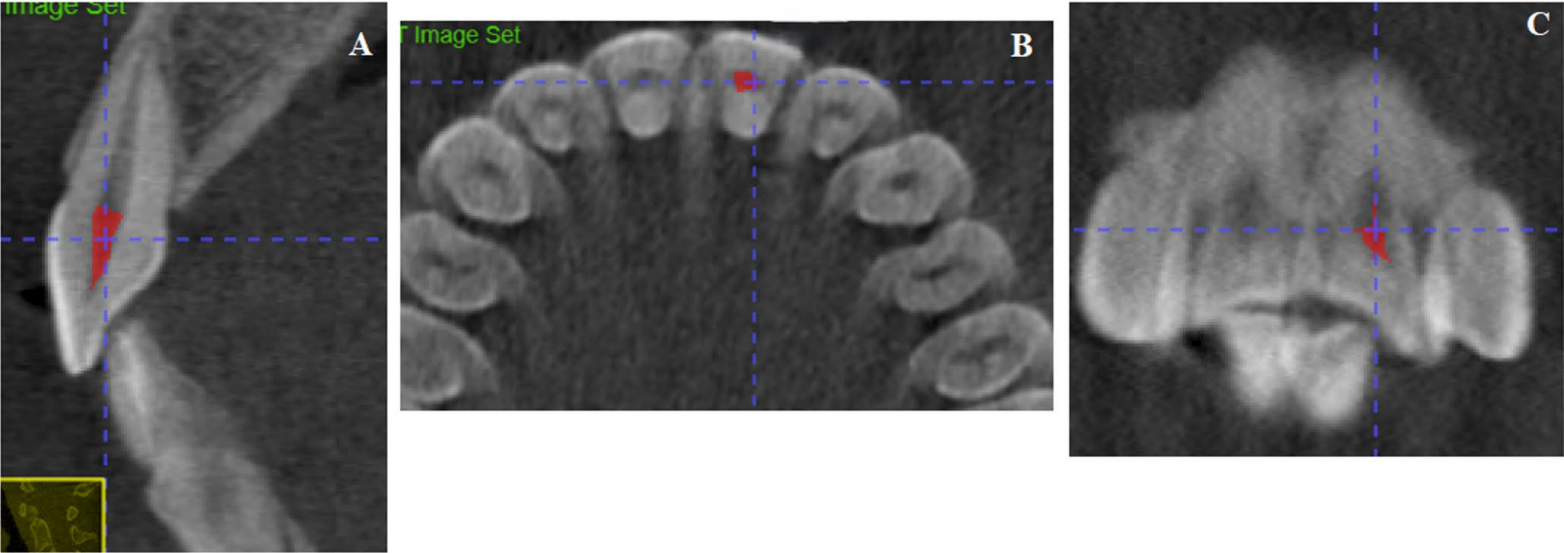
Maxillary Central incisor coronal pulp volume (MICPV). MICPV measured by tracing the pulp chamber within the crown in all three sections (sagittal-A, axial-B, and coronal-C), then using volume using ITK-SNAP software

*Maxillary incisor pulp chamber volume (MICPV)* measured by tracing the pulp chamber within the crown in all 3 sections (sagittal-A, axial-B, and coronal-C), then using volume using ITK-SNAP software (Fig. 5).

*Total pulp volume (TPV)* measured by tracing the whole pulp space of the tooth in all 3 sections (axial-A, coronal-B, and sagittal-C), then using volume and statistics in ITK-SNAP.

*Maxillary incisor crown height (MICH*) measured as the vertical distance from the incisal edge to the CEJ along the tooth’s long axis, in the sagittal view.

*Maxillary incisor crown width (MICW)* Maxillary incisor crown width was measured as the widest mesiodistal distance at the interproximal contact area of the crown, in the frontal view.

*Maxillary central incisor total length* measured from the incisal edge to the apex of the root along the tooth’s long axis, in the sagittal view.

### Statistical analysis

Statistical analysis was performed with the use of the Statistical Package for Social Science (SPSS) computer software (SPSS 23, SPSS Inc., NY, USA). Descriptive statistics were calculated for all the measured variables for each group. The Shapiro-Wilk test was applied to assess the normality of numeric data, and the results indicated that the data were not normally distributed. Differences between the studied groups were evaluated using the Kruskal-Wallis and Mann-Whitney nonparametric tests. The association between the PCV and the studied variables was analyzed using linear regression analyses. The level of significance was set at *P* ≤ 0.05.

## Results

Intra-class correlation coefficients ranged from 0.881 to 0.924 for inter-observer and from 0.813 to 0.913 for intra-observer, reflecting excellent reliability.

## First molars’ pulp chambers dimensions and volumes

### Based on the vertical skeletal pattern

Table 1 presents the means, standard deviations, Kruskal-Wallis test statistics, and P values.

**Table 1:** Comparison of mean values (± SD) for pulp chamber width (PCW-F, PCW-R), pulp chamber height (PCH-Mesial, PCH-Distal, PCH-Middle), pulp chamber volume (PCV), enamel thickness (En-Thick-Mesial, En-Thick-Distal), dentin thickness (Den-Thick-Mesial, Den-Thick-Distal), mesiodistal crown width (MDCW-F, MDCW-R), and crown height (CH-Mesial, CH-Distal) in maxillary, mandibular, and total first molars across vertical facial patterns: short face (SF), average face (AF), and long face (LF). Group differences tested using Kruskal–Wallis test with pairwise comparisons (SF-AF, SF-LF, AF-LF). Statistically significant values are shown in bold. NS = not significant

	Maxillary molars				Mandibular molars				Total molars (maxillary and mandibular)			
	SF (*n* = 76)	AF (*n* = 179)	LF (*n* = 102)	X^2^, *P* value	SF (*n* = 72)	AF (*n* = 156)	LF (*n* = 92)	X^2^, *P* value	SF (*n* = 148)	AF (*n* = 335)	LF (*n* = 194)	X^2^, *P* value
PCW-F	2.72 (0.55)	2.87 (0.60)	3.07 (0.66)	SF-AF (−1.69, *P* = 0.091) SF-LF (−3.54, *P* = 0.000) AF-LF (−2.46, *P* = 0.014)	4.11 (0.54)	4.09 (0.57)	4.42 (0.63)	SF-AF (−0.28, *P* = 0.978) SF-LF (−3.23, *P* = 0.001) AF-LF (−3.90, *P* = 0.000)	3.40 (0.88)	3.44 (0.85)	3.71 (0.93)	SF-AF (−0.48, *P* = 0.629) SF-LF (−3.01, *P* = 0.003) AF-LF (−3.11, *P* = 0.002)
PCW-R	3.84 (0.61)	3.95 (0.65)	4.01 (0.69)	NS (*P* = 0.196)	4.97 (0.90)	4.92 (0.70)	5.09 (0.71)	NS (*P* = 0.227)	4.39 (0.95)	4.40 (0.82)	4.52 (0.88)	NS *P* = 0.159)
PCH-Mesial	3.76 (0.83)	4.00 (0.75)	4.01 (0.71)	NS (*P* = 0.057)	3.58 (0.86)	3.81 (0.79)	3.92 (0.73)	SF-AF (−1.78, *P* = 0.075) SF-LF (−2.88, *P* = 0.004) AF-LF (−1.51, *P* = 0.131)	3.67 (0.85)	3.91 (0.78)	3.97 (0.72)	SF-AF (−2.82, *P* = 0.005) SF-LF (−3.59, *P* = 0.000) AF-LF (−1.26, *P* = 0.209)
PCH-Distal	2.99 (0.74)	3.21 (0.65)	3.33 (0.65)	SF-AF (−2.19, *P* = 0.029) SF-LF (−2.87, *P* = 0.004) AF-LF (−1.09, *P* = 0.277)	2.73 (0.76)	2.91 (0.69)	3.05 (0.78)	SF-AF (−1.58, *P* = 0.115) SF-LF (−2.49, *P* = 0.013) AF-LF (−1.27, *P* = 0.203)	2.86 (0.76)	3.07 (0.69)	3.20 (0.73)	SF-AF (−2.61, *P* = 0.009) SF-LF (−3.85, *P* = 0.000) AF-LF (−1.81, *P* = 0.070)
PCH- Middle	2.17 (0.63)	2.37 (0.61)	2.39 (0.64)	SF-AF (−2.59, P = **0.010**) SF-LF (−2.54, P = **0.011**) AF-LF (−0.24, *P* = 0.808)	1.67 (0.58)	1.92 (0.59)	1.91 (0.66)	SF-AF (−2.83, *P* = 0.005) SF-LF (−2.23, *P* = 0.026) AF-LF (−0.40, *P* = 0.687)	1.92 (0.65)	2.16 (0.64)	2.16 (0.69)	SF-AF (−3.61, *P* = 0.000) SF-LF (−3.18, *P* = 0.001) AF-LF (−0.10, *P* = 0.917)
PCV	6.79 (3.24)	7.75 (2.88)	8.22 (3.05)	SF-AF (−2.83, *P* = 0.005) SF-LF (−3.57, *P* = 0.000) AF-LF (−1.24, *P* = 0.216)	6.63 (3.24)	7.77 (2.76)	8.08 (2.90)	SF-AF (−3.67, *P* = 0.000) SF-LF (−3.82, *P* = 0.000) AF-LF (−0.59, *P* = 0.558)	6.72 (3.23)	7.76 (2.82)	8.14 (2.97)	SF-AF (−4.59, *P* = 0.000) SF-LF (−5.21, *P* = 0.000) AF-LF (−1.28, *P* = 0.201)
En-Thick-Mesial	1.44 (0.19)	1.49 (0.19)	1.54 (0.20)	SF-AF (−1.53, *P* = 0.127) SF-LF (−3.33, *P* = 0.001) AF-LF (−2.38, *P* = 0.012)	1.53 (0.21)	1.58 (0.26)	1.65 (0.25)	SF-AF (−1.06, *P* = 0.290) SF-LF (−3.28, *P* = 0.001) AF-LF (−2.78, *P* = 0.005)	1.49 (0.20)	1.53 (0.23)	1.60 (0.23)	SF-AF (−1.71, *P* = 0.088) SF-LF (−4.54, *P* = 0.000) AF-LF (−3.63, *P* = 0.000)
En-Thick-Distal	1.48 (0.18)	1.51 (0.20)	1.61 (0.28)	SF-AF (−0.98, *P* = 0.327) SF-LF (−3.36, *P* = 0.001) AF-LF (−3.03, *P* = 0.002)	1.54 (0.24)	1.59 (0.28)	1.63 (0.29)	NS (*P* = 0.084)	1.51 (0.21)	1.55 (0.24)	1.62 (0.29)	SF-AF (−1.48, *P* = 0.138) SF-LF (−3.95, *P* = 0.000) AF-LF (−3.15, *P* = 0.000)
Den-Thick-Mesial	2.82 (0.54)	2.80 (0.51)	2.68 (0.50)	NS (*P* = 0.130)	2.78 (0.59)	2.75 (0.52)	2.75 (0.50)	NS (*P* = 0.938)	2.80 (0.56)	2.78 (0.52)	2.71 (0.50)	NS *P* = 0.332)
Den-Thick-Distal	2.82 (0.57)	2.85 (0.53)	2.89 (0.51)	(*P* = 0.745)	2.86 (0.51)	2.91 (0.53)	2.90 (0.59)	NS (*P* = 0.983)	2.84 (0.54)	2.88 (0.53)	2.89 (0.55)	NS *P* = 0.885)
MDCW-F	7.96 (0.63)	7.86 (0.81)	8.16 (0.90)	NS (*P* = 0.134)	9.15 (0.69)	8.86 (0.68)	9.29 (0.86)	SF-AF (−2.62, *P* = 0.009) SF-LF (−1.03, *P* = 0.303) AF-LF (−4.07, *P* = 0.000)	8.54 (0.88)	8.33 (0.90)	8.70 (1.05)	SF-AF (−1.93, *P* = 0.053) SF-LF (−1.20, *P* = 0.232) AF-LF (−3.56, *P* = 0.000)
MDCW-R	10.17 (0.98)	10.22 (1.06)	10.50 (1.17)	NS (*P* = 0.060)	10.79 (1.01)	10.54 (0.78)	10.97 (0.90)	SF-AF (−2.40, *P* = 0.017) SF-LF (−0.92, *P* = 0.359) AF-LF (−3.70, *P* = 0.000)	10.47 (1.03)	10.37 (0.95)	10.72 (1.07)	SF-AF (−1.02, *P* = 0.306) SF-LF (−2.31, *P* = 0.021) AF-LF (−3.91, *P* = 0.000)
CH-Mesial	7.77 (1.22)	7.95 (1.08)	8.11 (1.01)	NS (*P* = 0.215)	7.70 (1.04)	7.88 (1.08)	8.13 (1.02)	NS (*P* = 0.051)	7.74 (1.14)	7.92 (1.08)	8.12 (1.02)	SF-AF (−1.44, *P* = 0.150) SF-LF (−2.92, *P* = 0.004) AF-LF (−1.95, *P* = 0.051)
CH-Distal	7.40 (1.19)	7.73 (1.14)	7.87 (1.15)	SF-AF (−1.91, *P* = 0.056) SF-LF (−2.50, *P* = 0.012) AF-LF (−0.94, *P* = 0.345)	6.85 (0.99)	7.23 (1.10)	7.41 (1.12)	SF-AF (−2.50, *P* = 0.012) SF-LF (−3.16, *P* = 0.002) AF-LF (−1.07, *P* = 0.285)	7.13 (1.13)	7.49 (1.15)	7.65 (1.15)	SF-AF (−3.15, *P* = 0.002) SF-LF (−3.95, *P* = 0.000) AF-LF (−1.34, *P* = 0.180)

#### Pulp chamber width (PCW)

In both jaws, PCW-F was significantly greater in the LF group compared to SF (*P* < 0.01) and AF (*P* < 0.05). At the pulp chamber roof, no significant differences were found (*P* > 0.05).

#### Pulp chamber height (PCH)

In the maxilla, mesial PCH showed no significant difference (*P* = 0.057), while middle and distal PCH were significantly shorter in the SF group (*P* < 0.05). In the mandible, SF subjects had significantly reduced PCH across all regions compared to LF, and in the middle region compared to both AF and LF (*P* < 0.05).

#### Pulp chamber volume (PCV)

PCV was significantly lower in SF individuals in both jaws compared to AF and LF groups (*P* < 0.01).

#### Enamel thickness (En-thick)

In the maxilla, LF subjects had significantly greater mesial and distal En-Thick than SF and AF (*P* < 0.05). In the mandible, mesial En-Thick was also greater in LF (*P* < 0.01), while distal differences were not significant (*P* > 0.05).

#### Dentine thickness (Den-thick)

In both the maxilla and the mandible, Den-thick on the mesial and distal sides showed no significant differences across groups (*P* > 0.05).

#### Mesiodistal crown widths (MDCW)

In the maxilla, MDCW showed no significant differences group differences at either the pulp chamber floor or roof (*P* > 0.05), while in the mandible, it was significantly reduced in the AF group compared to the SF and LF groups at both levels (*P* < 0.01).

#### Crown height (CH)

Distal CH was significantly shorter in SF compared to LF in both jaws (*P* < 0.05). No significant differences were found on the mesial side (*P* > 0.05).

### Based on the maxillary and mandibular molar relationships (CFM and CCM)

The means, standard deviations (SD), Mann-Whitney test statistics for the differences between studied groups, and P values are shown in Table 2.

**Table 2:** Comparison of mean values (± SD) for pulp chamber width (PCW-F, PCW-R), pulp chamber height (PCH-Mesial, PCH-Distal, PCH-Middle), pulp chamber volume (PCV), enamel thickness (En-Thick-Mesial, En-Thick-Distal), dentin height (Den-Thick-Mesial, Den-Thick-Distal), mesiodistal crown width (MDCW-F, MDCW-R), and crown height (CH-Mesial, CH-Distal) in maxillary and mandibular, and total first molars between cusp-to-fossa (CFM) and cusp-to-cusp (CCM) occlusal relationship groups. Group differences tested using Mann–Whitney U test; results shown as Z score (P value). Statistically significant values are shown in bold

	Maxillary molars			Mandibular molars			Total molars (maxillary and mandibular)		
	CFM (*n* = 228)	CCM (*n* = 129)	Z (*P* value)	CFM (*n* = 213)	CCM (*n* = 107)	Z (*P* value)	CFM (*n* = 441)	CCM (*n* = 236)	Z (*P* value)
PCW-F	2.91 (0.65)	2.86 (0.55)	−0.45 (*P* = 0.653)	4.21 (0.59)	4.15 (0.61)	−1.35 (*P* = 0.177)	3.54 (0.90)	3.44 (0.86)	−1.46 (*P* = 0.143)
PCW-R	3.99 (0.66)	3.87 (0.63)	−1.34 (*P* = 0.179)	4.99 (0.68)	4.94 (0.88)	−0.19 (*P* = 0.848)	4.47 (0.84)	4.36 (0.93)	−1.94 (*P* = 0.053)
PCH-Mesial	4.04 (0.76)	3.81 (0.75)	−2.02 **(P=0.043)**	3.86 (0.76)	3.67 (0.86)	−1.38 (*P* = 0.169)	3.95 (0.76)	3.74 (0.80	−2.33 **(P= 0.020)**
PCH-Distal	3.29 (0.70)	3.02 (0.63)	−3.46 **(P=0.001)**	2.99 (0.73)	2.75 (0.72)	−2.06 **(P=0.039)**	3.15 (0.73)	2.90 (0.69)	−3.64 **(P=0.000)**
PCH- Middle	2.38 (0.68)	2.26 (0.52)	−1.61 (*P* = 0.107)	1.88 (0.64)	1.82 (0.56)	−0.51 (*P* = 0.608)	2.14 (0.70)	2.06 (0.58)	−1.11 (*P* = 0.268)
PCV	7.89 (3.04)	7.31 (3.03)	−1.88 (*P* = 0.060)	7.68 (2.95)	7.41 (2.98)	−0.85 (*P* = 0.397)	7.79 (2.99)	7.36 (3.00)	−1.93 (*P* = 0.053)
En-Thick-Mesial	1.52 (0.20)	1.45 (0.19)	−3.63 **(P=0.000)**	1.60 (0.26)	1.56 (0.23)	−1.56 (*P* = 0.120)	1.56 (0.23)	1.50 (0.21)	−3.73 **(P=0.000)**
En-Thick-Distal	1.56 (0.24)	1.50 (0.21)	−2.53 **(P=0.011)**	1.61 (0.29)	1.55 (0.24)	−2.03 **(>P=0.042)**	1.58 (0.26)	1.53 (0.22)	−3.30 **(P=0.001)**
Den-Thick-Mesial	2.74 (0.53)	2.82 (0.49)	−1.51 (*P* = 0.132)	2.77 (0.52)	2.73 (0.55)	−0.61 (*P* = 0.540)	2.75 (0.53)	2.78 (0.51)	−0.74 (*P* = 0.460)
Den-Thick-Distal	2.77 (0.52)	2.99 (0.52)	−3.81 **(P=0.000)**	2.87 (0.51)	2.95 (0.60)	−0.74 (*P* = 0.459)	2.82 (0.52)	2.98 (0.56)	−3.13 **(P=0.002)**
MDCW-F	8.05 (0.79)	7.82 (0.82)	−1.87 (*P* = 0.061)	9.12 (0.74)	8.90 (0.78)	−2.68 **(P=0.007)**	8.57 (0.94)	8.31 (0.97)	−3.17 **(P=0.002)**
MDCW-R	10.41 (0.98)	10.07 (1.22)	−2.39 **(P=0.017)**	10.77 (0.82)	10.62 (1.02)	−1.23 (*P* = 0.217)	10.59 (0.92)	10.32 (1.16)	−2.70 **(>P=0.007)**
CH-Mesial	8.06 (1.07)	7.78 (1.13)	−2.12 **(P=0.034)**	8.00 (1.03)	7.73 (1.11)	−2.20 **(P=0.034)**	8.04 (1.05)	7.75 (1.12)	−2.96 **(P=0.003)**
CH-Distal	7.78 (1.16)	7.55 (1.18)	−1.65 (*P* = 0.099)	7.28 (1.08)	7.02 (1.10)	−1.87 (*P* = 0.062)	7.54 (1.15)	7.31 (1.17)	−2.25 **(P=0.025)**

#### Maxillary molars

CCM subjects had significantly lower values than CFM in mesial and distal PCH, En-Thick, MDCW-R at the pulp chamber roof, and CH (*P* < 0.05). However, distal dentin height was significantly higher in CCM (*P* < 0.05).

#### Mandibular molars

CCM subjects showed significantly greater values in distal PCH, MDCW at the floor (*P* < 0.01), mesial CH, and distal En-Thick (*P* < 0.05).

## Maxillary central incisor

### Based on the vertical skeletal pattern

Table 3 shows the means, standard deviations, test statistics, and P values for pulp dimensions and volumes.

**Table 3:** Comparison of mean values (± SD) for crown dimensions and pulp parameters of the maxillary central incisors across vertical skeletal patterns: short face (SF), average face (AF), and long face (LF). Variables include crown height (MICH), crown width (MICW), total tooth length (TTL), pulp chamber height (MIPCH), pulp chamber volume (MIPCV), and total pulp volume (TPV). Group differences were analyzed using the Kruskal-Wallis test (**X**^**2**^**)** with pairwise comparisons via Mann-Whitney U test (Z). Statistically significant differences are shown in bold. NS: not significant

	SF (*n* = 80)	AF (*n* = 185)	LF (*n* = 109)	X^2^ (*P* value)	Z (*P* value)
MICH	10.83 (1.10)	10.73 (1.19)	10.93 (1.09)	NS (*P* = 0.237)	-
MICW	9.94 (0.82)	9.92 (0.63)	9.97 (1.18)	NS (*P* = 0.769)	-
TL	23.29 (2.34)	23.57 (2.55)	24.16 (2.54)	NS (*P* = 0.057)	-
MIPCH	5.75 (0.93)	5.88 (1.07)	6.11 (1.22)	(7.20, P = **0.027**)	SF-AF (1.30, *P* = 0.193) SF-LF (2.57, P = **0.010**) AF-LF (1.84, *P* = 0.066)
MIPCV	2.68 (1.21)	3.17 (1.32)	3.68 (1.82)	17.38, (**0.000**)	SF-AF (2.90, P = **0.004**) SF-LF (4.02, P = **0.000**) AF-LF (2.05, P = **0.041**)
TPV	4.67 (1.78)	5.61 (2.04)	6.12 (2.60)	(19.79, P < **0.000**)	SF-AF (3.71, P = **0.000**) SF-LF (4.21, P = **0.000**) AF-LF (1.19, *P* = 0.233)

#### Maxillary incisors dimensions

No significant differences were observed in CH, width, or total tooth length across the groups (*P* > 0.05).

#### Pulp chamber height

LF group had a significantly greater mean PCH than the SF group (*P* < 0.05); other comparisons were not significant (*P* > 0.05).

### Pulp chamber volume

SF group had significantly lower PCV than both AF (*P* < 0.004) and LF (*P* < 0.001); LF also differed significantly from AF (*P* = 0.041).

### Maxillary central incisor total pulp volume

SF group had the smallest total pulp volume compared to LF and AF (*P* < 0.001).

### Based on the maxillary and mandibular central incisor teeth contact (Table 4)

**Table 4:** Comparison of mean values (± SD) for crown and pulp measurements of the maxillary central incisors between subjects with and without incisal contact. Variables include crown height (MICH), crown width (MICW), total tooth length (TTL), pulp chamber height (MIPCH), pulp chamber volume (MIPCV), and total pulp volume (TPV). Data were analyzed using the Mann-Whitney U test (Z). No statistically significant differences were found (*P* > 0.05)

	Incisors not contacting (*n* = 145)	Incisors are contacting (*n* = 229)	Z (*P* value)
**MICH**	10.85 (0.95)	10.78 (1.26)	−0.84 (*P* = 0.403)
**MICW**	9.95 (1.08)	9.94 (0.69)	−0.85 (*P* = 0.394)
**TTL**	23.89 (2.37)	23.56 (2.61)	−0.97 (*P* = 0.330)
**MIPCH**	6.03 (0.99)	5.84 (1.15)	−1.72 (*P* = 0.086)
**MIPCV**	3.39 (1.57)	3.11 (1.46)	−1.69 (*P* = 0.090)
**TPV**	5.75 (2.35)	5.45 (2.14)	−1.12 (*P* = 0.264)

None of the studied maxillary incisors’ variables showed significant differences between the 2 studied groups (anterior teeth with no opposing tooth contact vs. anterior teeth with opposing tooth contact).

### Associations between PCV and the studied variables (Table 5)

**Table 5:** Linear regression analysis identifying predictors of pulp cavity volume in maxillary first molars and maxillary central incisors. The dependent variable is pulp cavity volume. Statistically significant values are shown in bold

Molars	Unstandardized Coefficients		Standardized Coefficients	t	*P* value
	B	Std. Error	Beta		
Age	− 0.154	0.014	− 0.387	−10.800	**0.000**
Gender	0.957	0.216	0.157	4.433	**0.000**
Vertical pattern (1 = short, 2 average, 3 long face)	0.245	0.150	0.058	1.638	0.102
Molars code	0.805	0.186	0.347	4.332	**0.000**
Molars interdigitation	−2.133	0.503	− 0.340	−4.242	**0.000**
Transverse buccal occlusion	0.599	0.289	0.073	2.075	**0.038**
Jaw	− 0.175	0.208	− 0.029	− 0.842	0.400
En-Thick-Mesial	1.110	0.602	0.085	1.844	0.066
En-Thick-Distal	− 0.506	0.533	− 0.042	− 0.950	0.343
Den-Thick-Mesial	0.459	0.218	0.080	2.105	**0.036**
Den-Thick-Distal	− 0.209	0.214	− 0.037	− 0.977	0.329
Incisors					
Age	− 0.091	0.010	− 0.426	−8.944	**0.000**
Gender	0.436	0.140	0.143	3.112	**0.002**
Vertical pattern (1 = short, 2 average, 3 long face)	0.308	0.103	0.144	2.990	**0.003**
Incisor codes	0.156	0.064	0.128	2.442	**0.015**
Overjet	0.071	0.041	0.097	1.714	0.087
Overbite	0.124	0.041	0.156	2.994	**0.003**
Teeth contact	− 0.109	0.157	− 0.035	− 0.699	0.485

Linear regression analysis identified six significant predictors for molar PCV: age (*P* < 0.001), gender (*P* < 0.001), molar relationship (*P* < 0.001), interdigitation type (CFM/CCM) (*P* < 0.001), crossbite presence (*P* = 0.038), and mesial cusp dentine thickness (*P* = 0.036).

For maxillary incisor PCV, five predictors were significant: age (*P* < 0.001), gender (*P* = 0.002), vertical pattern (*P* = 0.003), incisor relationship (*P* = 0.015), and overbite (*P* = 0.003).

## Discussion

This study is the first to assess PCV and crown dimensions of first molars and maxillary incisors across different skeletal and dental malocclusions using CBCT. Unlike earlier reports that mainly linked anterior open bite and occlusal hypofunction to enlarged pulp spaces [12, 13], our results expand this relationship by showing how vertical facial morphology and occlusal patterns influence pulp size and crown morphology. CBCT provided accurate 3D analysis [13–15], while ITK-SNAP enabled precise pulp segmentation [11, 16], validated for forensic age estimation [14, 17]. To control age-related pulp reduction from secondary dentin deposition [18], only subjects over 18 were included.

This study found consistently lower pulp chamber volume and height in short-faced subjects compared to average-faced and long-faced groups in both first molars and maxillary incisors, accompanied by reduced enamel thickness and crown height. These findings echo previous reports that individuals with stronger bite forces and more developed masticatory muscles exhibit greater secondary dentin deposition and faster functional enamel wear [7, 14, 19, 20]. In contrast, long-faced subjects displayed significantly larger PCV and greater enamel thickness, consistent with previous observations that hypofunctional teeth tend to retain wider pulp chambers and thinner dentinal walls [13]. This pattern likely reflects reduced occlusal stimulation, as LF individuals typically exhibit lower BF and more limited masticatory efficiency. Reduced mechanical stress may slow the rate of secondary dentin deposition, preserving a larger pulp chamber over time [21]. These findings corroborate earlier studies showing that occlusal hypofunction results in larger pulp spaces, while functional hyperactivity, as seen in SF cases, leads to pulp reduction [12], [22], [23]. Interestingly, dentinal thickness remained relatively stable across facial types, suggesting that enamel and pulp tissue are more sensitive indicators of functional load variation than dentin, except under extreme conditions. Clinically, this suggests that pulp size may serve as a functional marker; patients with long-face morphology may be more prone to pulp exposure during restorative procedures.

Comparisons between cusp-to-cusp and cusp-to-fossa molar interdigitation further emphasized the role of force distribution. Subjects with CCM interdigitation exhibited reduced PCH and enamel thickness but greater dentin thickness and larger crown dimensions. These patterns reflect the elevated functional stresses associated with premature contacts [24], encouraging compensatory secondary dentin deposition and crown morphology alterations [6, 21, 25]. Previous studies have suggested that Class III malocclusions, which often display cusp-to-cusp contacts, may be associated with higher BF due to increased occlusal stress [7, 26]. Conversely, others have reported lower BF in Class III subjects due to reduced occlusal efficiency and contact area [27].

In the current study, subjects with CCM molar relationships showed reduced PCV, likely reflecting the heightened occlusal stress and premature contacts associated with this pattern [6, 25]. By contrast, CFM interdigitation appeared to promote more favorable force distribution, mitigating localized stress and preserving pulp volume [28]. The present findings suggest a possible reconciliation: rather than force magnitude alone, the way forces are distributed across the occlusal surface plays a decisive role in determining pulp response. Clinically, this underscores the importance of achieving proper occlusal interdigitation to minimize long-term pulpal stress.

BF has a strong link to vertical craniofacial morphology but shows no significant correlation with sagittal patterns [14, 29], 30]. Posterior teeth in Class II and III cases often show compensatory inclinations; however, occlusal force and contact area are similar across vertical types within Class I, II, and III malocclusions [31].

Another notable finding was the observation of larger PCV in individuals with posterior crossbites. At first glance, this result seems unexpected as crossbites are frequently associated with asymmetric muscle activity, premature contacts, and localized occlusal stress [32]. However, one possible explanation is that reduced overall bite force and inefficient load distribution in crossbite cases may actually lead to hypofunctional loading of certain teeth, thereby preserving larger pulp chambers [20]. Importantly, the current study did not differentiate between unilateral and bilateral crossbites, which may have contributed to the variability in results and warrants further investigation. This suggests that clinicians should not assume uniform functional overloading in crossbite cases and should consider individual variability in functional adaptation.

Regression analysis confirmed that age and gender also significantly affect pulp size, in line with earlier reports [23, 24, 33, 34]. Age demonstrated a negative correlation with PCV, confirming that secondary dentin deposition continues throughout adulthood and progressively reduces pulp space [23, 24]. Gender differences were also apparent, with males displaying larger pulp chambers, a finding consistent with earlier reports that attribute pulp size differences to genetic and hormonal influences [33, 34]. These demographic factors should be considered when using pulp size for forensic age estimation [35]. Although PCV can predict chronological age [34], its accuracy varies by tooth type, as other studies found weak correlations, suggesting additional contributing variables [35, 36].

Although descriptive comparisons indicated significantly reduced PCV in SF individuals, regression analyses revealed that vertical skeletal pattern did not retain significance as an independent predictor of molar pulp volume after adjusting for other variables. This suggests that the influence of vertical morphology on molars may be indirect, mediated by occlusal force distribution and age-related changes. Interestingly, the vertical pattern remained significant for incisor PCV, consistent with earlier reports that reduced functional loading in open bite and low BF cases is associated with preserved pulp space [7, 13, 19]. Thus, incisors appear more directly sensitive to vertical loading differences, while molars reflect a more complex interplay of variables.

This study has several limitations. Although all participants were adults over 18 years of age, individual variation in secondary dentin deposition may still have influenced pulp volume measurements. The sample was restricted to Jordanian adults, which may limit the generalizability of the findings to other populations. Furthermore, bite force was not directly measured but rather estimated from previous studies on individuals with comparable skeletal patterns, which may not fully reflect the actual functional forces in this cohort.

Future research should include larger and more diverse samples to improve the generalizability of findings across different populations and ethnic groups. In addition, combining three-dimensional craniofacial analysis with clinical measurement of bite force and tooth contact in a longitudinal study design could better link facial pattern to pulp morphology and age-related PCV changes.

## Conclusions

This study shows that PCV and dimensions of maxillary and mandibular first molars and maxillary central incisors vary significantly across occlusal and skeletal patterns. SF exhibited the smallest PCV, and CCM was associated with reduced molar PCV. Age and gender were also significant factors, where PCV decreased linearly with age and was generally larger in males. Interestingly, the presence of a posterior crossbite was associated with increased molar pulp volumes. These results support considering vertical pattern and interdigitation during endodontic access planning and radiographic interpretation. Clinically, tailoring access preparation to the patient’s anatomical pattern can reduce complications and improve outcomes.

## Clinical relevance

These results emphasize the multifactorial influences on pulp size, shaped by demographic, anatomical, and functional factors. This highlights the need for personalized assessments in endodontic, restorative, or forensic planning [14, 35]. Understanding these anatomical variations helps clinicians prevent over- or under-preparation during endodontic procedures and avoid misdiagnosis of pulp size on radiographs. In SF individuals, where the pulp chamber is relatively small, careful access cavity preparation using magnification and adequate illumination is essential to avoid iatrogenic perforation. In LF individuals, where the pulp chamber is larger, meticulous caries removal is necessary to prevent accidental pulp exposure. Awareness of these anatomical differences also improves radiographic interpretation, reducing the risk of under- or overestimating pulp size when skeletal and dental morphology is not considered.

## Data Availability

No datasets were generated or analysed during the current study.
